# Adipose-derived mesenchymal stem cell-loaded β-chitin nanofiber hydrogel promote wound healing in rats

**DOI:** 10.1007/s10856-021-06630-7

**Published:** 2022-01-20

**Authors:** Ying Liu, Yunen Liu, Mi Wu, Rufei Zou, Shun Mao, Peifang Cong, Mingxiao Hou, Hongxu Jin, Yan Zhao, Yongli Bao

**Affiliations:** 1 National Engineering Laboratory for Druggable Gene and Protein Screening, Northeast Normal University, Changchun, 130117 China; 2 Emergency Medicine Department of General Hospital of Northern Theater Command, Laboratory of Rescue Center of Severe Wound and Trauma PLA, Shenyang, 110016 China; 3 Jihua Laboratory, Foshan, 528200 China

## Abstract

Because of stem cells are limited by the low efficiency of their cell homing and survival in vivo, cell delivery systems and scaffolds have attracted a great deal of attention for stem cells’ successful clinical practice. β-chitin nanofibers (β-ChNF) were prepared from squid pens in this study. Fourier transform infrared spectroscopy, X-ray diffraction and scanning electron microscopy proved that β-ChNFs with the diameter of 5 to 10 nm were prepared. β-ChNF dispersion became gelled upon the addition of cell culture medium. Cell culture experiments showed that β-ChNFs exhibited negligible cytotoxicity towards ADSCs and L929 cells, and it was found that more exosomes were secreted by the globular ADSCs grown in the β-ChNF hydrogel. The vivo experiments of rats showed that the ADSCs-loaded β-ChNF hydrogel could directly cover the wound surface and significantly accelerate the wound healing and promote the generation of epithelization, granulation tissue and collagen. In addition, the ADSCs-loaded β-ChNF hydrogel clearly regulated the expressions of VEGFR, α-SMA, collagen I and collagen III. Finally, we showed that ADSCs-loaded β-ChNF hydrogel activated the TGFβ/smad signaling. The neutralization of TGFβ markedly reduced Smad phosphorylation and the expressions of TIMP1, VEGFR and α-SMA. Taken together, these findings suggest that ADSCs-loaded β-ChNF hydrogel promises for treating wounds that are challenge to heal via conventional methods.

**Figure:**
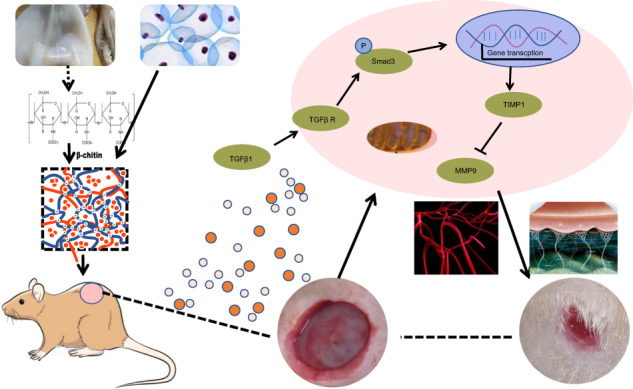
Graphical abstract

Graphical abstract

## Introduction

As a soft tissue that covers the entire surface area of the body, skin is mainly responsible for protecting various tissues and organs from physical, mechanical, chemical and pathogenic microorganisms [1]. Wound, as one of the most commonly encountered healthcare issue, could induce significant distress to patients by providing a portal of entry into the body for pathogens, and their appropriate management is critical for the wellbeing of affected individuals [2, 3]. Wound healing includes the granulation tissue formation, scar tissue formation and other synergistic effects. During the healing process, numerous distinct cells, especially stem cell are activated, recruited into the wound, and secreted various growth factors and cytokines [4–6]. With the development of regenerative medicine, stem cells are becoming new strategies for effective wound treatment [7, 8].

The abundant supply of fat tissue as well as the ease of isolation, proliferation and secretion of growth factors related to wound healing, makes adipose-derived mesenchymal stem cells (ADSCs) an ideal cell type for non-healing wound treatment. ADSCs can be differentiated into different lineages and secrete paracrine factors to initiate the tissue regeneration process [9]. Currently, intravenous infusion and local injection are the major routes for ADSC administration, which are limited by the low efficiency of their cell homing and survival in vivo during clinical application [10]. Therefore, the efficacy of cell therapy significantly depends on whether cellular carriers could successfully deliver therapeutic cells to target tissues and sites, as well as the surrounding microenvironment [11, 12].

By incorporating therapeutic cells, cell delivery systems provide a cell-friendly environment that can be placed at the target site, and it showed that ADSCs combined with different scaffolds could noticeably improve wound healing [13]. To date, cell delivery systems and scaffolds have attracted a large amount of attention, especially cellular scaffolds composed of various synthetic and natural polymers. Typically be prepared under mild processing conditions, hydrogel is a kind of polymer network system with a certain shape. They have soft viscoelastic properties and are similar in function and structure to extracellular matrix (ECM). Hydrogels have been widely used in the tissue engineering and regenerative medicine fields because of their various properties as well as supporting the incorporation of therapeutic cells [14, 15]. Chitin is the second most abundant polysaccharide in nature after cellulose and is widely found in the shells of shrimps, crabs, insects and other crustaceans, as well as the cell walls of fungi and bacteria. It has already been used in suture line, artificial dialysis membrane, tissue engineering and wound repair [16]. Nanofibrous structures of chitin nanofibers are excellent candidates as wound dressing materials: their porous structure simulates the fibrous structure of natural extracellular matrix; facilitates cell seeding; allows diffusion of nutrients, oxygen and waste; and can absorb wound exudates, to prevent excessive dehydration and bacterial infection from the wound [17]. Chitin exists as typically two different forms in nature, namely α and β. Formed by two symmetrically and alternately antiparallel chitin chains, α-chitin has higher degree of intermolecular hydrogen bonding and a more compact and stable structure. While β-chitin has intrasheet hydrogen-bonding by parallel chains with weaker intermolecular interactions between the chains and higher chemical reactivity. Kangquan Shou et al has found that α-chitin nanofibers hydrogel could affect bone marrow mesenchymal stem cell (BMSC) differentiation and exhibit better viability of the cells than local injection of BMSC in wound treatments [18]. However, the characteristics of β-chitin nanofibers in ADSCs cell culture, and their effect on exosome secretion and wound healing remain to be further studied.

In this work, β-chitin nanofibers (β-ChNF) were obtained from squid cartilage, and used to prepare scaffold carriers for stem cells application. Following toxicity evaluation and the determination of successful stem cells growth in the scaffold, rat wound models were established to evaluate the wound healing effect and mechanism of the dressing. The results provide new perspectives for the application of stem cells by using β-ChNF hydrogel as the scaffold for full-thickness cutaneous wound healing.

## Materials and methods

### Materials

Squid pens were obtained from a local fish market, China. Hydrochloric acid, sodium hydroxide and acetic acid were purchased from Sinopharm Chemical Reagent Co., Ltd., China. ADSCs (MUBMD-01001) and adipose-derived mesenchymal stem cell medium (MUBMD-03011-440) were purchased from Cyagen Biosciences, China. L929 (BFN60805937) cells were provided by Cobioer, China. Masson’s staining kit was purchased from Sigma, USA.

### Preparation of β-chitin

β-chitin was prepared from squid pens through demineralization and deproteinization steps. Briefly, 50 g of powdered squid pen was washed and dried, and dispersed in 750 mL of 0.1 mol/L hydrochloric acid solution for 20 h for demineralization. After washing with D.I. water to neutrality, squid pens were further treated with 750 mL of 4 wt% sodium hydroxide solution for 10 h at 80 °C for deproteinization and washed with D.I. water at a resistivity of 18.2 MΩ•cm to neutrality.

### Gelation of β-chitin nanofiber (β-ChNF) dispersion

A β-ChNF dispersion was prepared by adding the purified β-chitin to D.I. water at a concentration of 0.3 wt%, acidified to pH 3 with acetic acid, and treated by the ultrasonic homogenizer (Scientz JY92-IIDN, China) in an ice-water bath for 6 min at 19.5 kHz and 300 W. Gelation of the β-ChNF dispersion occurred when culture medium was added to the dispersion at a ratio of 3:1 β-ChNF dispersion:medium. The formation of β-chitin was confirmed by Fourier transform infrared (FTIR) spectroscopy (Agilent Technologies Cary 630 spectrometer). The sample’s crystallinity was characterized by X-ray diffraction (XRD; Max-2500PC, Rigaku D, Japan). The morphology of the β-ChNF dispersion before and after gelation was observed through scanning electron microscopy (SEM, inspect F50, FEI). The rheological characterization of the β-ChNF dispersion with and without added culture medium was conducted via a rheometer (HAAKE MARS40, Germany).

### Cell culture and cytotoxicity

ADSCs were cultured in adipose-derived mesenchymal stem cell medium that contained 10% fetal bovine serum, 1% penicillin-streptomycin (T180125G001), and 1% glutamine (T180516G001). For the hydrogel cell culture, cell medium was added to the β-ChNF solution at a 3:1 volume ratio, respectively, and allowed to gel for 15 min at room temperature, then 1 × 10^6^ ADSCs were added. L929 cells were cultured in MEM cell medium (CPB50009) containing 10% horse serum, 1% penicillin-streptomycin (CPB50027) and 1% glutamine (CPB50028). According to ISO 10993 part 5 guidelines (ISO document 10993, 1992), the cytotoxicity of the β-ChNF hydrogel was evaluated by an extraction test and 3-(4,5-dimethylthiazol-2-yl)-2,5-diphenyltetrazolium bromide (MTT) assay of ADSCs and L929 cells. The cells were seeded in 96-well plates and cultured in 37 °C for 24 h with 5% CO_2_. The medium in hydrogel group was replaced with the extracts. The control group was treated with normal culture media. After culturing for a further 12, 24, or 48 h, 20 μL of MTT (5 mg/mL in PBS) was added to each well. 4 h later, the medium containing MTT was replaced with 150 μL of dimethyl sulfoxide (DMSO). After incubation for 15 min with agitation, the absorbance of the DMSO solution at 490 nm was measured using a plate reader (BIO-RAD iMark). Cell growth in the scaffold was observed via an inverted phase contrast microscope (TS100, Nikon, Japan). Exosome secretion was detected by transmission electron microscopy (G2 spititi FEI, Tecnai) and nanoparticle tracking analysis (ZetaView_Particle Metrix, ZetaView PMX 110, Particle Metrix). During the nanoparticle tracking analysis, the sample pool was cleaned with deionized water and the instrument was calibrated with polystyrene microsphere (110 nm). After that, 1X PBS buffer (Biological Industries, Israel) was used to clean the sample pool and the sample was diluted with 1X PBS buffer (BI, Israel) to be checked.

### Animals

Sprague–Dawley rats (6–8 weeks) were purchased from Liaoning Changsheng Biotechnology Co., Ltd. and were kept in their cages maintaining at 22 ± 3 °C with a humidity of 45–60%. They were all allowed free access to food and water. After acclimation, all rats were randomly divided into the control, hydrogel, ADSCs and ADSCs+hydrogel groups. Animal experiments were approved by the Ethics Committee of the General Hospital of the Northern Theater Command.

### Wound healing activity

The full-thickness defect wound model was established on the basis of a procedure used in a previous report [19]. Circular full-thickness cutaneous wounds were created and carefully observed 0, 2, 4, 7, 8 and 10 days post-surgery. The wound was photographed and the area was measured by Image J (National Institutes of Health, USA). The wound healing rate was calculated as follows: wound healing rate (%) = (A0 − At)/ A0 × 100%.

### Histological examination

Ten days after surgery, part of the rat skin samples were harvested and fixed in 10% formaldehyde. For histological staining, 5-μm-thick sections were sliced and stained using hematoxylin and eosin (H&E) staining. To assess the amount of generated collagen, sections were stained with masson’s staining kit (Sigma, USA). Image J software was used to detect the percentage of the collagen staining area (blue) in the total area. Immunofluorescence staining was carried out to localize VEGFR and α-SMA. Briefly, skin tissues were dewaxed and the endogenous peroxidase was inactivated. After antigen retrieval, the samples were blocked with BSA, incubated with primary antibody and a fluorescent secondary antibody.

### Western blot

The protein for western blot analysis was extracted from skin tissues and concentration was determined using a BCA protein assay kit (Cat No: FD2001, Hangzhou Fude Biological Technology Company, China). The primary antibody included VEGFR (1:1000, ab39638, Abcam, UK), α-SMA (1:1000, ab124964, Abcam, UK), collagen I (1:1000, ab270993, Abcam, UK), collagen III (1:1000, ab7778, Abcam, UK), TGFβ1 (1:2000, ab215715, Abcam, UK), Smad3 (1:2000, ab40854, Abcam, UK), Smad2 (1:2000, ab40855, Abcam, UK), P-smad3 (phospho T179, 1:2000, ab74062, Abcam, UK), P-smad2 (phospho S467, 1:2000, ab280888, Abcam, UK), TIMP1 (1:2000, ab216432, Abcam, UK), GAPDH (1:4000, 2118, Cell Signaling Technology, Boston, USA) and Anti-rabbit secondary antibody (1:4000, ab6721, Abcam, UK). Proteins were visualized through a Clarity TM Western ECL Substrate (170–5061; Bio-Rad Laboratories Inc., USA) and a Tanon 5200 fully automatic chemiluminescence image analysis system (Tanon Science and Technology Co. Ltd., Shanghai, China).

### Blockade of TGFβ1

One day after treatment with ADSCs-loaded β-ChNF hydrogel, we hypodermic injected a neutralizing antibody (MAB 1835, R&D Systems, Minneapolis, MD) in a single dose of 400 μg/kg to a group of rats. Five days after the injection, the protein expression of TGFβ1, P-smad3, P-smad2, Smad3, Smad2, TIMP1, VEGFR and α-SMA in rats were further detected by western blot.

### Statistical analysis

All data were analyzed by SPSS 22.0 (IBM, NY, USA). Quantitative data were expressed as mean ± standard deviation. The two-tailed paired Student’s *t* test was used for group comparisons. *P* values < 0.05 were considered statistically significant. The correlation analysis between the concentration of VEGFR and the number of blood vessels was conducted by pearson test.

## Results

### Characterization of the porous chitin nanofiber hydrogel

**Fig. 1:**
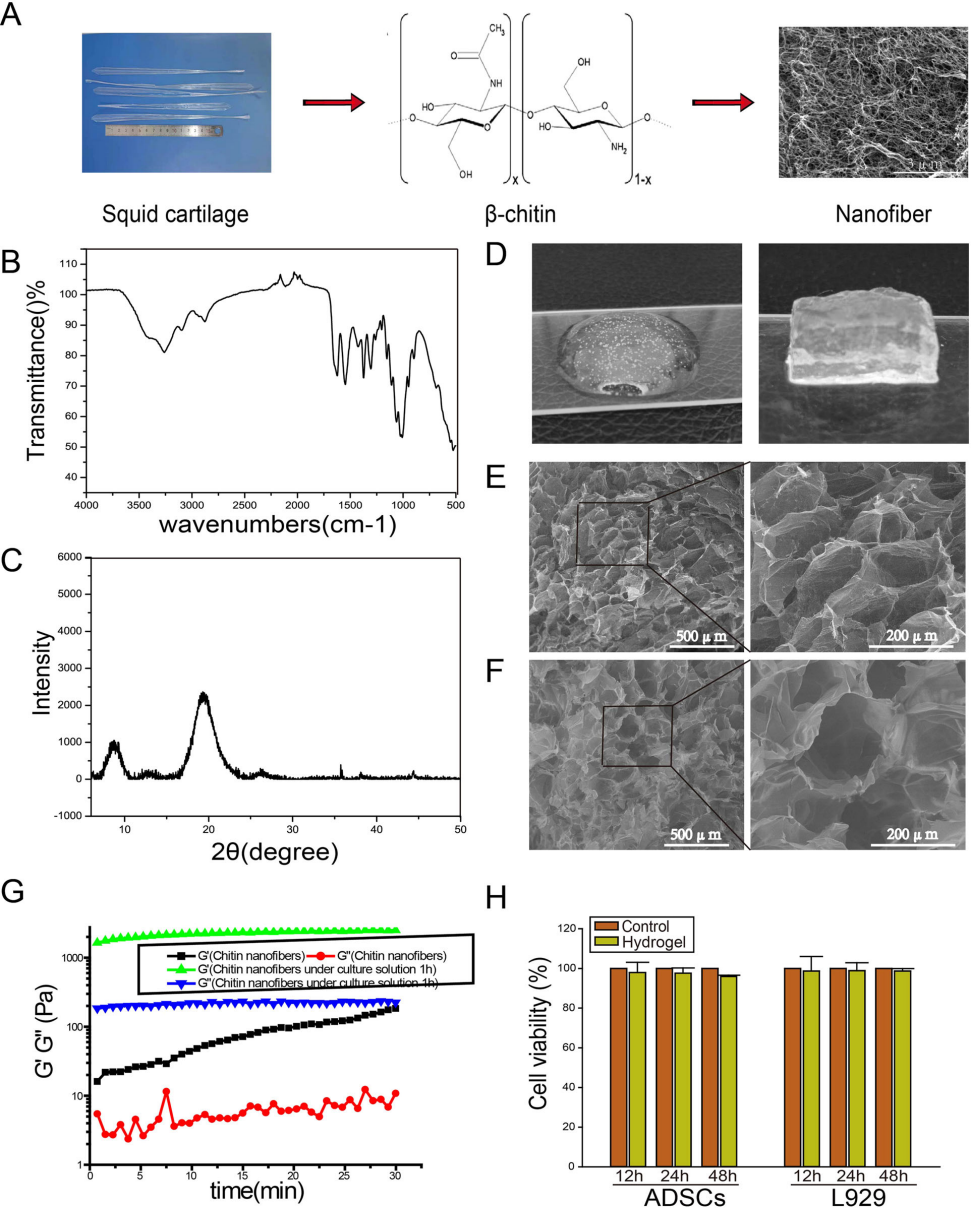
The characterization of β-chitin nanofibers hydrogel. **A** The sketch map of β-chitin nanofibers preparated from squid cartilage. **B** FTIR spectra and (**C**) XRD profile suggested a β-chitin structure. **D** Representative digital photographs of the β-chitin nanofibers hydrogel before and after the reaction with cell culture medium. **E** SEM image of the chitin hydrogel. **F** SEM image of the β-chitin hydrogel in the presence of culture solution. **G** Storage modulus (G′) and loss modulus (G″)-strain dependence of chitin nanofibers and chitin nanofibers in the presence of culture solution. **H** The extracts of β-chitin membrane incubated with ADSCs and L929 cells shown no cytotoxicity

Figure 1A shows the preparation process of β-chitin nanofiber with the degree of deacetylation of about 10% and a uniform width of 5–10 nm. To identity the physicochemical structure of the as-prepared chitin nanofiber, FTIR and XRD measurements have been carried out. As shown in Fig. 1B, the characteristic peaks of chitin nanofibers occurred at 3434 cm^−1^, 3270 cm^−1^, which could be assigned to the stretching vibration of O-H and N-H, respectively [20, 21]. In particular, the adsorption bands involving 1628 cm^−1^ (amide I) and 1550 cm^−1^ (amide II) suggested that a β-chitin structure was prepared from squid pen [22, 23]. As shown in XRD pattern (Fig. 1C), the main diffraction peaks of chitin nanofibers appeared at 8.6° (020 plane) and 19.6° (110 plane), further indicating the crystal structure of β-chitin [21]. Figure 1D and E shows the hydrogel before and after the addition of cell culture medium, respectively. The addition of cell culture medium initiated gel formation. Figure 1F, G shows SEM images of the chitin nanofiber and the morphological characteristics in presence of cell culture medium, respectively. The intermediate pore size of the hydrogels was ~200 μm. Figure 1H shows the gelling kinetics based on measuring G′ and G″ as a function of time. Both G’ and G” of the hydrogel increased significantly when cell culture medium was added, indicating a stronger hydrogel was formed. Figure 1I shows the results of the MTT assay, the viabilities of ADSCs and L929 cells cultured in the chitin membrane were more than 95%. These in vitro cytotoxicity results indicated that β-chitin had low cell toxicity and was applicable in cell culture.

### Preparation of ADSC-loaded β-ChNF hydrogel

**Fig. 2:**
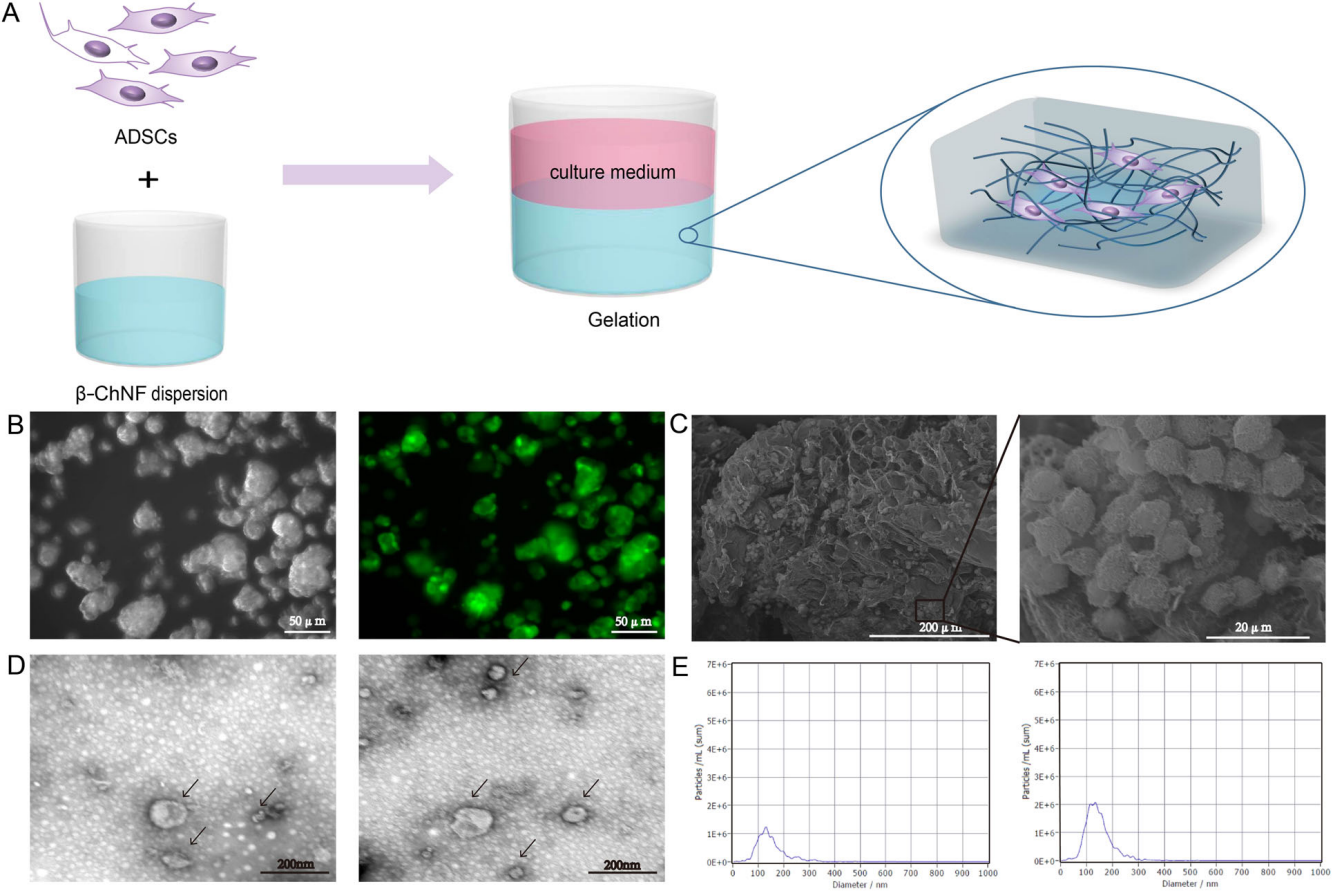
Growth of ADSCs in β-chitin hydrogel. **A** The stepwise diagram for preparation of ADSCs-loaded β-ChNF hydrogel. **B** Globular ADSCs grown in the β-chitin hydrogel scaffold, and the microexamination proved the growth of GFP-labeled ADSCs. **C** SEM images of the ADSCs-loaded β-ChNF hydrogel. **D** Transmission electron microscopy proved the secretion of exosomes in normal or β-chitin hydrogel culture (Left, the control group. Right, the hydrogel group). **E** Nanoparticle tracking analysis of exosomes in normal or β-chitin hydrogel culture (Left, the control group. Right, the hydrogel group)

β-ChNF hydrogel dispersion (500 μL) and C57BL/6 mouse ADSCs medium (1500 μL) were added to an ultra-low attachment 24-well plate. After 15 min, the medium was discarded and 1000 μL of C57BL/6 mouse ADSCs medium containing 1 × 10^6^ ADSCs was added (Fig. 2A). The microstructure of ADSCs-loaded β-ChNF hydrogel is shown in Fig. 2. Microscopy revealed that the ADSCs grew as globules (Fig. 2B) and the GFP-labeled ADSCs exhibited green fluorescence under a fluorescence microscope (Fig. 2C). The cell distribution was observed by scanning electron microscopy. The ADSCs-loaded β-ChNF hydrogel exhibited a highly porous and interconnected interior structure that supported cell growth (Fig. 2D, E). Furthermore, transmission electron microscopy was used to detect exosomes in the cell supernatant (Fig. 2F, G). Nanoparticle tracking analysis showed that there were more ADSC exosomes in the hydrogel cell culture than those in the control cell culture (Fig. 2H, I).

### ADSC-loaded β-ChNF hydrogels significantly increased the wound healing rate

**Fig. 3:**
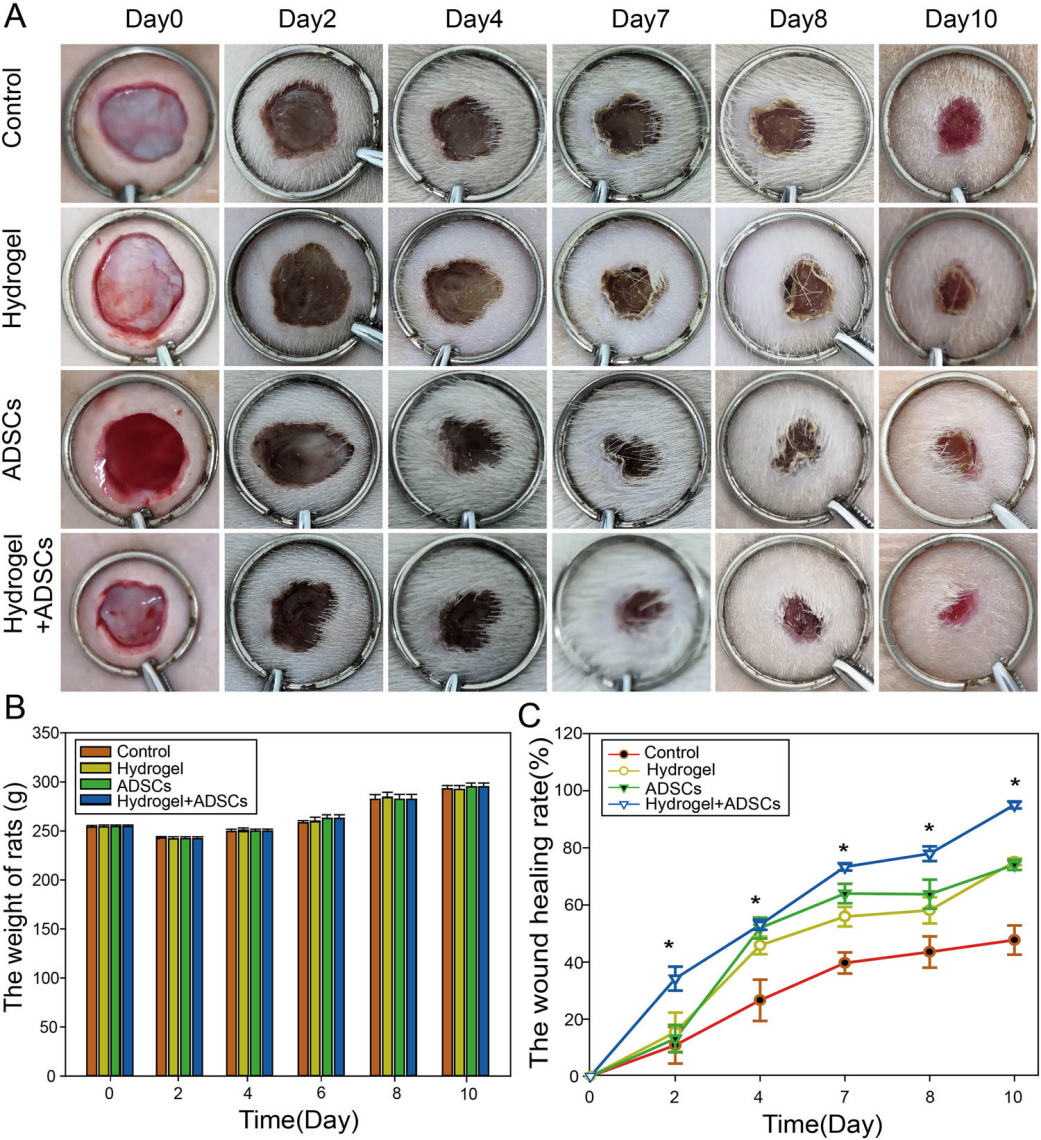
ADSCs-loaded β-ChNF hydrogel increased the wound healing rate in rats. **A** Photographs of wounds closure from 0 to 10 d after surgery. **B** Weight shown no changes during the treatment period. **C** The wound healing rate of full-thickness wounds for each treatment group. ^*^P < 0.05, vs. the control group

Rats in each group were in good condition. There was no significant difference in the body weight of rats among the groups. Two days after surgery, the wounds in all groups were dry, and no obvious infection, edema or inflammatory exudation was observed in the wounds or surrounding skin tissue. Seven days after surgery, the wound in the control group was hard, while the texture of the wounds in the hydrogel and ADSCs+hydrogel groups remained soft. In addition, the wounds in the ADSCs+hydrogel group were markedly smaller than those in the control, ADSCs or hydrogel groups. Image J software was used to measure the wound healing rate of the full-thickness wounds in each group. The results showed: compared with the control, ADSCs and hydrogel groups; the ADSCs-loaded β-ChNF hydrogel significantly increased the wound healing rate (Fig. 3, *P* < 0.05). Seven days after surgery, the wound healing rate of the ADSCs+hydrogel group was nearly 70%.

### ADSC-loaded β-ChNF hydrogels improved the pathological changes of wound in rats

**Fig. 4:**
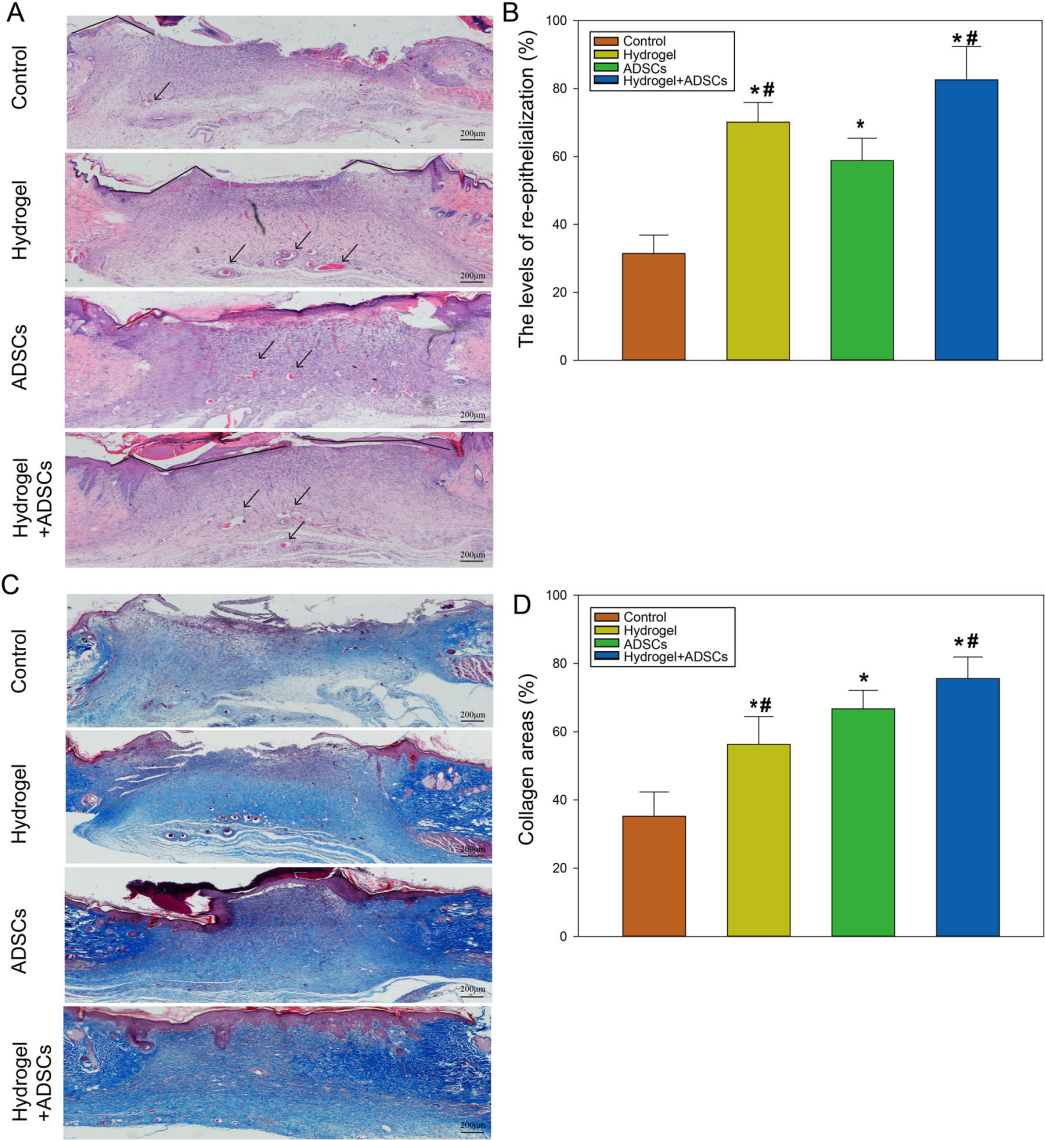
ADSCs-loaded β-ChNF hydrogel accelerated wound healing and collagen deposition in rats. **A** Representative H&E staining images of each group. The red arrows indicated new blood vessels. **B** Image J software was used to measure the levels of re-epithelialization of the full-thickness wounds in each group. ^*^P < 0.05, vs. the control group; ^#^*P* < 0.05, vs. the ADSCs group; ^&^*P* < 0.05, vs. the ADSCs+hydrogel group. **C** Representative modified Masson staining images of each group. **D** Image J software was used to measure the collagen deposition of the full-thickness wounds in each group. ^*^P < 0.05, vs. the control group; ^#^*P* < 0.05, vs. the ADSCs group; ^&^*P* < 0.05, vs. the ADSCs+hydrogel group

Histological examination showed poor granulation, incomplete healing, no epithelium formation and fewer capillaries in the control group; bleeding, necrosis, and scab were still present in the ADSCs and hydrogel groups; compared with the control group, the healing was significantly increased, the epithelialization rate was higher, and the granulation tissue and capillary formation were increased in the hydrogel, ADSCs or ADSCs+hydrogel group; compared with the hydrogel or ADSCs group, the epithelialization rate in the ADSCs+hydrogel group was also increased (Fig. 4A, *P* < 0.05); these were consistent with the above wound healing results. Furthermore, the hydrogel group rats showed better epithelialization than ADSCs group mice (Fig. 4A, *P* < 0.05). Modified Masson staining showed that the amount of collagen deposited 10 d after surgery in the hydrogel, ADSCs or ADSCs+hydrogel group was markedly higher than that in the control group; when compared with the ADSCs group, the collagen area in the ADSCs+hydrogel group was also increased (Fig. 4B, *P* < 0.05). These data suggested that ADSCs-loaded β-ChNF hydrogel showed the combined effects of hydrogels and ADSCs to accelerate wound healing in rats. ADSCs-loaded β-ChNF hydrogel took the advantage of hydrogel by accelerating epithelialization, and at the same time took the advantage of stem cell therapy by increasing collagen production.

### ADSC-loaded β-ChNF hydrogels accelerated collagen deposition and angiogenesis

**Fig. 5:**
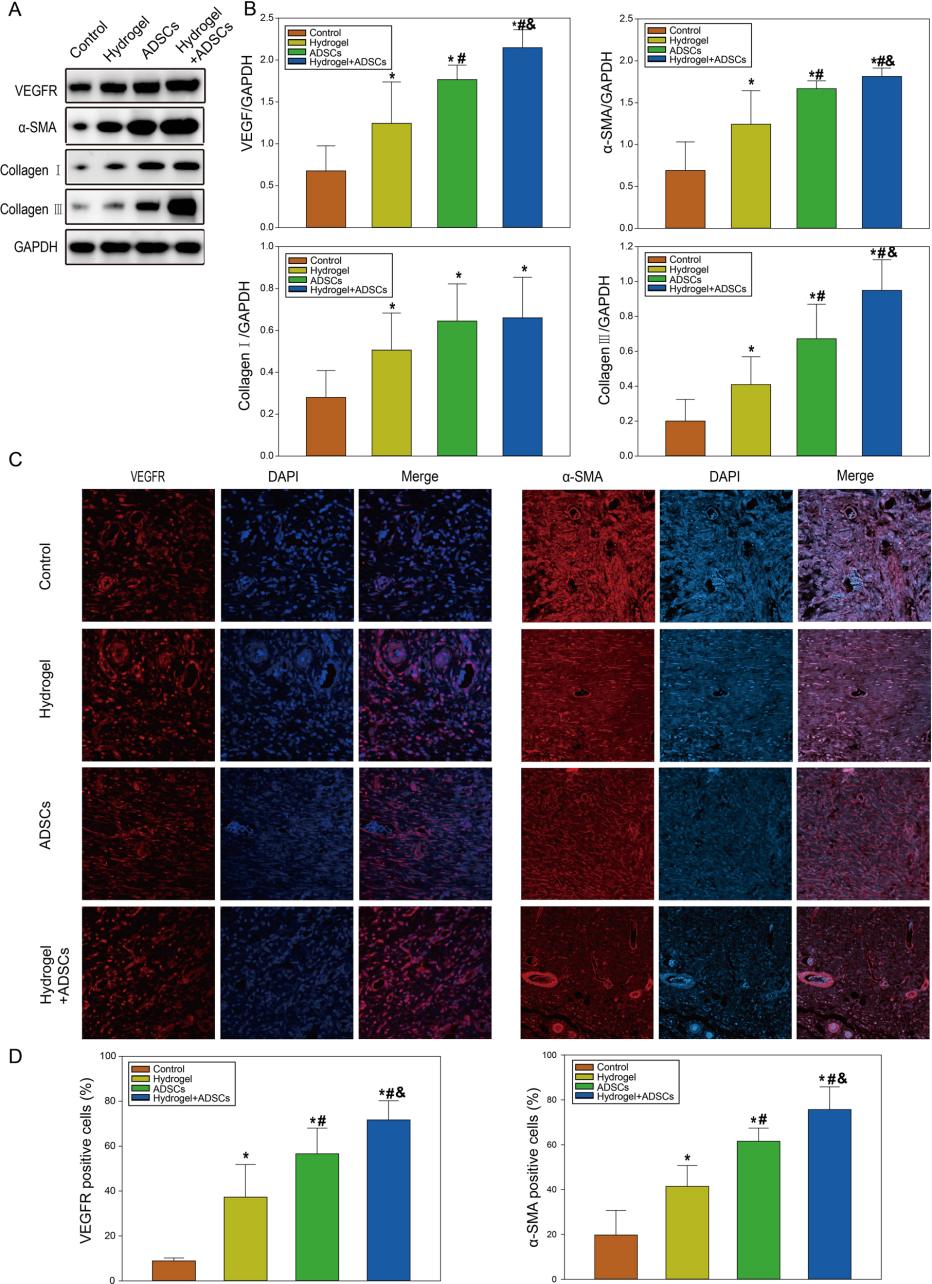
ADSCs-loaded β-ChNF hydrogel accelerated angiogenesis and collagen deposition. **A** Representative western blot images of VEGFR, α-SMA, collagen I, collagen III, and GAPDH. **B** Quantitative analysis of VEGFR, α-SMA, collagen I and collagen III. Results are expressed as the mean ± SD. ^*^P < 0.05, vs. the control group; ^#^*P* < 0.05, vs. the ADSCs group; ^&^*P* < 0.05, vs. the ADSCs+hydrogel group. **C** Representative immunofluorescent staining images showing the effect of ADSCs-loaded β-ChNF hydrogel on VEGFR and α-SMA expression. **D** The quantitative analysis of VEGFR, α-SMA. ^*^P < 0.05, vs. the control group; ^#^*P* < 0.05, vs. the ADSCs group; ^&^*P* < 0.05, vs. the ADSCs+hydrogel group

Western blot showed that the expressions of VEGFR, α-SMA, collagen I and collagen III of the hydrogel, ADSCs or ADSCs+hydrogel group were significantly higher than those of the control group 10 d after surgery; ADSCs-loaded β-ChNF hydrogel accelerated the expression of VEGFR and α-SMA when compared with hydrogel or ADSCs treatment; the ADSCs group mice also showed the higher expression of VEGFR and α-SMA than the hydrogel group mice (*P* < 0.05). Furthermore, ADSCs-loaded β-ChNF hydrogel group mice also showed higher expression of collagen III than ADSCs mice (Fig. 5A, B, *P* < 0.05). This indicated that ADSCs-loaded β-ChNF hydrogel promoted angiogenesis and collagenation in wound healing. ADSCs-loaded β-ChNF hydrogel was better for the expression of collagen III than hydrogel, which was good for preventing scar formation in wound healing. Immunofluorescence staining showed that the expression of VEGFR and α-SMA in the ADSCs+hydrogel group was significantly higher than that of the control group 10 d after surgery (Fig. 5C, *P* < 0.05). The number of blood vessels was positive correlation with the VEGFR concentration (*r* = 0.8499).

### ADSC-loaded β-ChNF hydrogels regulated the TGFβ/smad signaling pathway

**Fig. 6:**
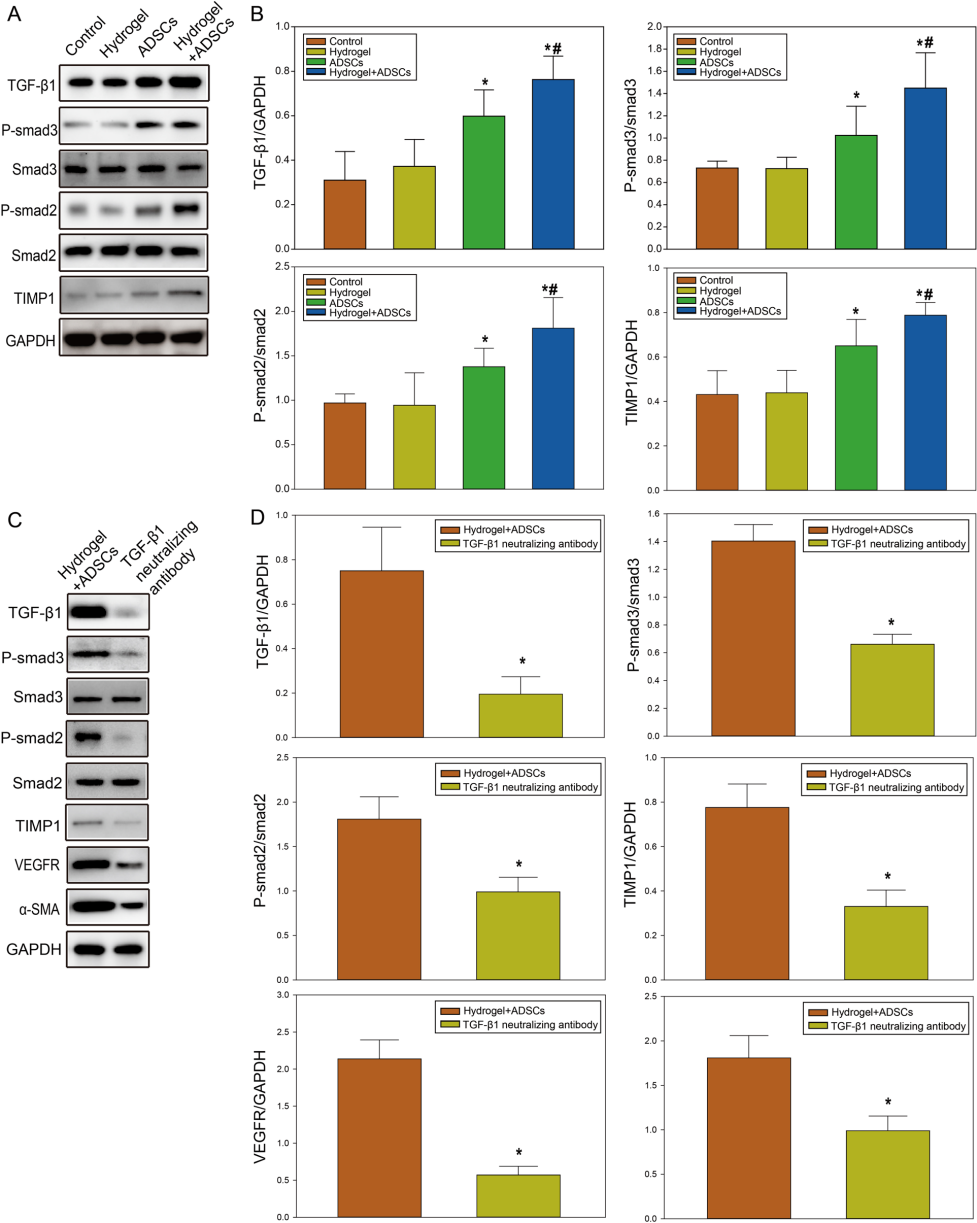
ADSCs-loaded β-ChNF hydrogel regulated the TGFβ/smad3 signaling pathway. **A** Representative western blot images of TGFβ1, P-smad3, smad3, P-smad2, smad2, TIMP1, and GAPDH. **B** Quantitative analysis of TGFβ1, P-smad3, smad3, P-smad2, smad2, and TIMP1. Results are expressed as the mean ± SD. ^*^P < 0.05, vs. the control group; ^#^*P* < 0.05, vs. the ADSCs group; ^&^*P* < 0.05, vs. the ADSCs^+^hydrogel group. **C** Representative western blot images of TGFβ1, P-smad3, smad3, P-smad2, smad2, TIMP1, and GAPDH after treatment with neutralizing antibody. **D** Quantitative analysis of TGFβ1, P-smad3, smad3, P-smad2, smad2, and TIMP1 after treatment with neutralizing antibody. Results are expressed as the mean ± SD. ^*^P < 0.05, vs. the control group; ^#^*P* < 0.05, vs. the ADSCs group; ^&^*P* < 0.05, vs. the ADSCs+hydrogel group

TGFβ can be regulated by mesenchymal stem cells and result in the phosphorylation of Smad2 and Smad3. These will trigger target gene transcription to play an active role in the wound healing process, collagenation as well as neoangiogenesis. To further clarify the mechanism of ADSC-loaded β-ChNF hydrogel on the wound, we detected the expression of TGFβ/smad signaling pathway. Western blot showed that the expressions of TGFβ1, P-smad3, P-smad2 and TIMP1 in the ADSCs and ADSCs+hydrogel groups were significantly increased at 10 d after surgery than those of the control group (*P* < 0.05), while there was no significant difference in the hydrogel group (*P* > 0.05). The expressions of TGFβ1, P-smad3, P-smad2 and TIMP1 in the ADSCs+hydrogel group were higher than those of the ADSCs group (Fig. 6A, *P* < 0.05). To prove the rule of TGFβ/smad in wound healing, we further detected the expression of VEGFR and α-SMA after blockade of TGFβ1. The results showed that the expression of VEGFR and α-SMA in ADSCs+hydrogel group were lower after neutralizing antibody treatment (Fig. 6B, *P* < 0.05). These date suggested that ADSC-loaded β-ChNF hydrogel and ADSCs could regulate the TGFβ/smad signaling pathway to promote neoangiogenesis.

## Discussion

In nature, chitin exists as different allomorphic forms that vary in terms of their polymer chain structure and crystallinity. α-Chitin has a two-chain, antiparallel structure, and was the most abundant form of chitin found in insects and crustaceans, while the rarer β-chitin allomorph occurs in squid pens and some diatoms, and has a one-chain unit cell with a parallel-chain structure [24, 25]. Due to its good biocompatibility and high porosity similar to the skin’s natural extracellular matrix, β-chitin nanofibers are conducive to cell adhesion and growth [26]. In this study, the squid cartilage was made into β-ChNF dispersion. We found that the β-ChNF dispersion was gelled upon the addition of cell culture medium, which was beneficial to its successful application. The weaker hydrogen-bonding of the parallel-chain structure of β-chitin may account for its higher chemical reactivity. In addition, β-chitin has the unique feature of incorporating small molecules, including water, into its crystal lattice to form crystalline complexes [27, 28]. The unique structure of intramolecular hydrogen bonds might facilitate the combination of the active components of the culture medium to form a gel. The hydrogels exhibited a highly and interconnected porous structure, which was expected to have a high permeability for nutrients and to support cellular growth.

The cell culture experiments in this study further proved the properties of β-ChNFs hydrogel in cellular growth. In vitro tests have shown that β-ChNFs exhibited negligible cytotoxicity towards ADSCs and L929 cells, and the globular ADSCs were found in the β-ChNF hydrogel. The loose porous structure could be highly permeable to nutrients and promote the spread of cells seeded on the chitin nanofibrous matrices, as well as the formation of cell clusters that were tightly packed. Porous chitin nanofiber was therefore a suitable substrate for cell adhesion and supports stem cell self-renewal [29]. Furthermore, it was found that more exosomes were secreted by the ADSCs grown in the β-ChNF hydrogel. The secretion of exosomes is one of the ways that stem cells exert biological activity. Exosomes have a double-layer membrane rich in bioactive molecules, such as cholesterol, sphingolipids, ceramides, nucleic acids, proteins, carbohydrates and other molecules. They could target specific receptor cells and act as carriers for intercellular signal transduction [30]. In this study, ADSCs were located on the inner walls of the scaffold pores, proliferated and grew well in the three-dimensional porous structure. The viable cells were uniformly distributed in the β-ChNF hydrogel and more exosomes were efficiently secreted. These properties indicate that the support is a viable biomaterial for accelerating the wound healing, which can solve the problem of cell localization and survival.

Healing of open cutaneous wounds comprises a complex series of events, namely epithelialization, connective tissue deposition and contraction [31]. The existing methods for promoting wound healing have some problems such as limited effect or inconvenient use. Stem cell therapy has been shown to be effective in treating the chronic wound, and the scaffold for stem cell played an important role for its successful applications [32]. In this study, β-ChNF hydrogel was investigated as the scaffold of ADSCs. ADSCs-loaded β-ChNF hydrogel showed a positive effect in 2 days, and promoted rats wound healing in 10 days. β-ChNF hydrogel can provide a wet healing environment for wound healing. Good hydration is an important external factor that contributes to optimal wound healing, which can prevent undesirable scab formation as well as further dermal damage induced by desiccation [33]. The observed improvement in healing may be related to easier migration of epidermal cells over the moist wound surface rather than beneath a dry scab and the preservation of growth factors and proteinases present in fluid exudates that can then exert their potentiating effect on wound healing [34]. In addition to the above functions, the ADSCs-loaded β-ChNF hydrogel also allowed ADSCs to persist within the wound area and accelerated wound healing. ADSCs has been reported to enhance tissue regeneration via two different mechanisms, either by differentiating into skin cells or by secretion of paracrine factors [35]. It has been found that ADSCs can stimulate angiogenesis, epithelialization, and wound remodeling through paracrine secretion during wound repair. In rodent burn models, ADSCs modulated expression at the cellular level, specifically increased the expression of VEGF and collagens, which were related to microvessel formation and damage repair [36]. However, the ADSCs served to quell inflammation and stimulate angiogenesis was mostly applied by injections. The advantage of this experiment is that the ADSCs-loaded β-ChNF hydrogel could directly load on the wound surface and significantly accelerate wound healing by accelerating epitheliogenesis and promoting angiogenesis as well as collagen deposition. Furthermore, the better effects of ADSCs-loaded β-ChNF hydrogel during wound healing than ADSCs or hydrogel can be attributed to the healing properties of the ADSCs and also the three-dimensional environment of the hydrogel.

Collagens are the primary dermal matrix, which are responsible for the proliferation, tensile strength of skin and the formation of scars [13]. The ADSCs-loaded β-ChNF hydrogel could effectively secrete exosomes and accelerate angiogenesis and collagen deposition, while the specific mechanism required determination. TGFβ/ Smad signaling pathway is related to the collagen formation during damage repair. Therefore, we observed the change of TGFβ/ Smad signaling pathway in this study. It is reported that mesenchymal stem cell-derived exosomes microRNAs could adjust the TGF-β/smad pathway during wound healing, which is consistent with our results [37]. TGF-β/smad is involved in wound healing processes through neoangiogenesis [38]. TIMP1 plays a role in inhibiting MMP deposition and preventing ECM degradation. In addition, TIMP1 has been known to promote fibroblast proliferation and enhance new blood vessel formation [39–41]. This study showed that ADSCs-loaded β-ChNF hydrogel treatment up-regulated the expression of TIMP1. Therefore, the success of β-chitin in this application is related to the provision of a three-dimensional hydrogel microenvironment for ADSCs to promote angiogenesis through the TGFβ/smad signaling pathway.

## Conclusion

In this study, squid pens were used to prepare β-chitin nanofiber dispersion, which gelled upon the addition of cell culture medium and created an appropriate microenvironment for cell adhesion and exosome secretion. The prepared hydrogel showed advantages as ADSC support and was demonstrated to be an effective carrier material for stem cells in the treatment of wounds. The mechanism of this study revealed that in addition to playing the role of hydrogel to accelerate epithelialization, ADSCs-loaded β-ChNF hydrogel could also significantly promote angiogenesis and collagen deposition through the TGFβ/smad3 signaling pathway. Our study demonstrates the potential of clinical applications of the stem cell-based β-chitin nanofiber hydrogels, which might be promising to be applied in the healing process of acute and chronic wounds.

## Data Availability

The datasets generated during and/or analyzed during the current study are available from the corresponding author on reasonable request.
